# Gender Identity and Assignment Recommendations in Disorders of Sex Development Patients: 20 Years’ Experience and Challenges

**DOI:** 10.4274/jcrpe.galenos.2020.2020.0009

**Published:** 2020-11-25

**Authors:** Fatih Gürbüz, Murat Alkan, Gonca Çelik, Atıl Bişgin, Necmi Çekin, İlker Ünal, Ali Kemal Topaloğlu, Ünal Zorludemir, Ayşe Avcı, Bilgin Yüksel

**Affiliations:** 1 Çukurova University Faculty of Medicine, Department of Pediatric Endocrinology, Adana, Turkey; 2 Çukurova University Faculty of Medicine, Department of Pediatric Surgery, Adana, Turkey; 3 Çukurova University Faculty of Medicine, Department of Child Psychiatry, Adana, Turkey; 4 Çukurova University Faculty of Medicine, Department of Medical Genetics, Adana, Turkey; 5 Çukurova University Faculty of Medicine, Department of Forensic Medicine, Adana, Turkey; 6 Çukurova University Faculty of Medicine, Department of Biostatistics, Adana, Turkey

**Keywords:** Gender assignment, disorders of sex development, ambiguous genitalia, congenital adrenal hyperplasia

## Abstract

**Objective:**

Gender assignment in infants and children with disorders of sex development (DSD) is a stressful situation for both patient/families and medical professionals.

**Methods:**

The purpose of this study was to investigate the results of gender assignment recommendations in children with DSD in our clinic from 1999 through 2019.

**Results:**

The mean age of the 226 patients with DSD at the time of first admission were 3.05±4.70 years. 50.9% of patients were 46,XY DSD, 42.9% were 46,XX DSD and 6.2% were sex chromosome DSD. Congenital adrenal hyperplasia (majority of patients had 21-hydroxylase deficiency) was the most common etiological cause of 46,XX DSD. In 46,XX patients, 87 of 99 (89.7%) were recommended to be supported as a female, 6 as a male, and 4 were followed up. In 46,XY patients, 40 of 115 (34.8%) were recommended to be supported as a female, and 70 as male (60.9%), and 5 were followed up. In sex chromosome DSD patients, 3 of 14 were recommended to be supported as a female, 9 as a male. The greatest difficulty in making gender assignment recommendations were in the 46,XY DSD group.

**Conclusion:**

In DSD gender assignment recommendations, the etiologic diagnosis, psychiatric gender orientation, expectation of the family, phallus length and Prader stage were effective in the gender assignment in DSD cases, especially the first two criteria. It is important to share these experiences among the medical professionals who are routinely charged with this difficult task in multidisciplinary councils.

**What is already known on this topic?**

Gender assignment in disorders of sex development (DSD) patients is always very difficult, complex and demanding experience in the management for both families and clinicians, particularly in cases where the gender appropriate for the clinical diagnosis is incompatible with the psychological gender of the patient. Gender assignment councils must have an experienced and multidisciplinary approach.

**What this study adds?**

Here, we present 20 years of experience and challenges in gender assignment, the causes and clinical characteristics of patients with DSD. This study is the longest timeframe, is the most comprehensive and has the largest number of cases in terms of gender assignment recommendation and assessing the factors affecting gender assignment from Turkey.

## Introduction

According to Jost’s paradigm, the first sexual development stage begins with the identification of the chromosomal sex at the time of fertilization and is completed as a result of many biological process (1). Money et al. (2) added the theory of psychosexual development to this paradigm. This theory is influenced by hormonal and genetic status, environmental and psychosocial experiences, and social and parental behavior (3,4,5). Any defect occurring during this complicated process of sexual differentiation may lead to a discordant development of chromosomal, gonadal, and anatomical sex/phenotype and is defined as disorders of sex development (DSD) (6,7,8). DSD are a heterogeneous group of rare conditions which include various etiologies and presentations (9,10,11). The incidence of DSD is almost 1 in 4,500-5,500 (10,11,12).

The long-term physical, social and psychological outcomes of patients with DSD are still unclear. There are increasing concerns regarding early decisions about gender assignment in recent reports (13,14,15,16,17,18). Studies have been generally conducted regarding psychosexual and surgical outcomes in this group of patients (19,20,21,22). Gender assignment of a child with DSD is the most difficult and stressful condition for both the family and the clinician, especially in cases of ambiguous genitalia (6,23,24). Families will always want to know the actual gender of their DSD baby as soon as possible and give their baby a gender appropriate name. The primary goal in DSD is for gender identity to be consistent with the gender assigned (6). In this respect, a multidisciplinary approach is required for the diagnosis and treatment of DSD (25). Influencing factors to consider when debating gender assignment include medical diagnosis, external genital appearance, potential of fertility and sexuality, therapeutic and/or surgical intervention options, views and desires of the patients and their families, sociocultural factors, and the psychological gender development status of the child (26,27,28).

There is a multidisciplinary council to make gender assignment recommendations in DSD patients which, in our clinic, consists of pediatric endocrinology, pediatric surgery, pediatric psychiatry, medical genetics and forensic science specialists. Here, we present 20 years of experience at a single regional referral center in assistance with gender assignment in DSD patients.

## Methods

The purpose of this study was to investigate the results of gender assignment recommendations in children with DSD and the factors affecting these results in our clinic. In the present study, the file records of the 226 children with DSD admitted to the Department of Pediatric Endocrinology of Çukurova University between the years of 1999 and 2019 were reviewed. The clinical diagnosis of a DSD was supported by anatomical examination findings, gonadal and pelvic ultrasound, cytogenetic studies, determination of serum electrolytes, 17-hydroxyprogesterone levels, the ratio of testosterone-dihydrotestosterone (basal and hCG stimulated) and molecular genetic testing. 21-hydroxylase deficiency (21-OHD) (72 of 88), 11-beta-hydroxylase deficiency (6 of 6), 17-beta-hydroxysteroid dehydrogenase type 3 deficiency (4 of 4), Steroidogenic Acute Regulatory Protein *(STAR)* gene mutations (5 of 5), complete androgen resistance (8 of 9), incomplete androgen resistance (6 of 6), 5-alpha-reductase deficiency, (19 of 19), Leydig cell aplasia/hypoplasia (2 of 2), 17-alpha-hydroxylase deficiency, (1 of 1), DSS-AHC Region on Human X Chromosome (*DAX1*; also known as *NR0B1*) (2 of 2), *NR5A1 (SF1)* (2 of 2), Persistent Mullerian Duct syndrome (1 of 1), and Klinefelter syndrome (2 of 2) were diagnosed by cytogenetic studies and molecular genetic analyses. However, mixed gonadal dysgenesis, gonadal dysgenesis, ovotestis and Sertoli cell only syndrome were diagnosed by laparoscopy with gonadal biopsy, and molecular genetic testing. All the genetic testing was performed for diagnostic purposes after consent from the patients or child’s legal representative.

Laparoscopy and gonadal biopsy were performed in selected DSD patients for determination of gonadal histology. Cystoscopy was performed in order to examine urethra, uterus and uterine remnants.

Our center is the first, and the oldest and largest ‘Gender Evaluation Council’ in the region. This council consists of pediatric endocrinologists, pediatric surgeons, child psychiatrists, specialists in forensic medicine and a medical geneticist. Gender assignment recommendations were made by this council. The role of the council is to evaluate medical data, to conduct expert discussion, and to provide information and medical advice to the patient and/or family. The council ensures that ample time and opportunities are provided to patient and families for their questions, concerns, and counseling needs.

Exclusion criteria for this study were: DSD patients who did not need gender assignment (therefore not discussed in the council) such as Turner syndrome and isolated hypospadias. Written inform consent was obtained after the council from the parents or legal guardians of all the patients before participation. The study protocol was approved by the Ethics Committee of Çukurova University and performed in accordance with the ethical standards of the Declaration of Helsinki (ethical decision no: 452018.77/10).

Background clinical data obtained from medical file records included age at the time of first admission and meeting, reason for admission, genital examination findings, Prader stage, karyotype, diagnosis, psychiatric gender orientations, gender patient was being raised as, parents’ views and requests for the gender, number of council meetings held for each patient, and gender assigned. Although genital phenotype evaluation according to the Sinnecker classification is more appropriate for 46,XY DSD cases (29), all patients were evaluated via Prader classification in order to avoid confusion (30).

The patients were classified into three main groups on the basis of the karyotype of the affected individual, according to The Lawson Wilkins Pediatric Endocrine Society and the European Society for Paediatric Endocrinology consensus (8,9,31). These groups are: 46,XX DSD; 46,XY DSD; and sex chromosome DSD.

The psychological evaluation for gender orientation was based on psychiatric interview with children and according to Diagnostic and Statistical Manual of Mental Disorders-5 diagnostic criteria (32).

### Statistical Analysis

All analyses were performed using SPSS, version 20.0 statistical software package (IBM Inc., Armonk, NY, USA). Categorical variables were expressed as numbers and percentages, whereas continuous variables were summarized as mean and standard deviation (SD). Chi-square test was used to compare categorical variables between the groups. The normality of distribution for continuous variables was confirmed with the Shapiro-Wilk test. For comparison of continuous variables between two groups, the Student’s t-test or Mann-Whitney U test was used depending on the distribution being normal or non-parametric, respectively. For comparison of continuous variables between more than two groups, Kruskal Wallis test was used. Bonferroni adjusted Mann-Whitney U test was used for pairwise comparisons of groups. The statistical level of significance for all tests was considered to be 0.05.

## Results

A total of 226 patients were classified as 46,XY DSD (n=115, 50.9%), 46,XX DSD (n=97, 42.9%) or sex chromosome DSD (n=14, 6.2%) (Table 1). The mean±SD age at first admission of the patients was 3.05±4.70 (range 0-17.58) years.

Of the 226 patients, ambiguous genitalia (n=141, 62.4%) was the most frequent cause of admission for all three groups (Table 1).

When the diagnostic distribution of the patients was examined, congenital adrenal hyperplasia (CAH) was the most common cause of DSD. Among the 46,XX DSD (n=97) patients, 21-OHD was the most common (n=88, 90.7%) (Table 1). The most common cause amongst 46,XY DSD cases (n=115) was 5-alpha reductase deficiency (n=19, 16.5%). This was followed by complete androgen insensitivity syndrome (CAIS) and incomplete androgen resistance (PAIS) (total n=15, 13%). Forty-two (18.6%) of all cases had undetermined causes for DSD. The vast majority of these were 46,XY DSD cases (40/42, 95.2%) (Table 1, 2).

**Figure 1:**
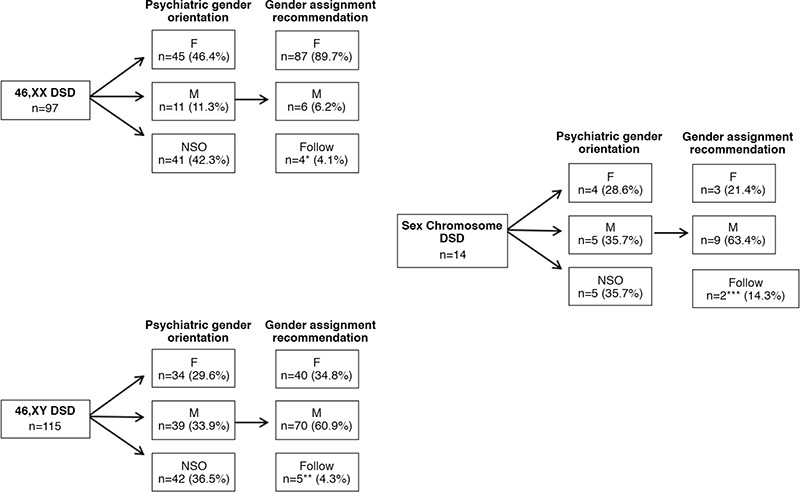
Gender orientations and gender assignment recommendations DSD: disorders of sex development, F: female, M: male, NSO: no sexual orientation *Two of the 3 cases with 46,XX related to 21-hydroxylase deficiency were raised as a male and their families insisted on an assignment recommendation as the male gender. The other one case had female gender orientation, but the family wanted to raise as the male gender. The remaining one 46,XX DSD patient had 11-OH deficiency, and raised as the male gender. Moreover, patient’s family wanted to raise as the male gender although the patient had menstrual bleeding. **For 2 cases with 46,XY DSD diagnosed with 5-alpha reductase deficiency a follow-up recommendation was made, who were raised as female gender instead of male gender by their parents. Families were persistently wanting for a female assignment to be made. The other two 46,XY DSD cases had a diagnosis of gonadal dysgenesis and had not yet developed a gender orientation. The one 46,XY DSD patient had 17-betahydroxysteroid dehydrogenase type 3 deficiency, was raised as a female and the family asked for a male gender assignment. ***The one Klinefelter syndrome case was raised as a female and her family wanted to raise as the female gender. The other one patient was mixed gonadal dysgenesis and had no gender orientation yet.

The psychiatric evaluation of cases showed that only about half of the 46,XX DSD patients had female and one in three of the 46,XY DSD patients had male gender orientation. In the sex chromosome DSD cases, female gender was 4/15 and male gender was 5/14 patients and 5/15 patients had no sexual orientation (Figure 1).

The median age of all cases was 1.90 (mean: 4.46±4.98, range 0.12-18.63) years at the time of the council meeting. For each of the categories 46,XX DSD, 46,XY DSD and sex chromosome DSD patients these median ages were 1.60 (mean: 3.20±3.92, range 0.12-18.56), 2.98 (mean: 5.49±5.41, range 0.13-18.38) and 1.67 (mean: 4.77±6.15, range 0.21-18.63) years, respectively (p=0.004). While 200 (88.5%) of 226 patients had gender assignment at the first council meeting, 26 patients (11.5%) had more than one council meeting of whom 18/26 were 46,XY DSD, six were 46,XX DSD and two were sex chromosomal DSD patients. It is notable that patients requiring more than one meeting were mostly 46,XY DSD cases.

The mean age intervals of presentation and being considered at the meeting for 46,XX DSD, 46,XY DSD, and sex chromosome DSD were 1.19±2.03 (range 0.06-10.96) years, 1.45±2.12 (range 0.03-11.97) years and 2.73±4.58 (range 0.02-15.08) years, respectively. It was found that, these intervals were not different according to the DSD diagnosis (p=0.113), Prader stage (p=0.949) and decision (p=0.062).

In 46,XY DSD patients, 40 of 115 (34.8%) were recommended to be assigned as a female gender (Figure 1). The female gender assignment recommendation in these cases was made for all of the CAIS, Leydig cell aplasia/hypoplasia, *STAR* gene mutations, 17-alpha hydroxylase and *DAX1 (NR0B1)* mutation cases according to the genetic diagnosis (Table 2).

Eleven of 226 cases (4.8%) were followed without a gender assignment (Figure 1). The common characteristic of all these cases who were not assigned a gender was that the family’s gender expectation was not compatible with chromosomal analysis, specific diagnosis, Prader stage and/or psychiatric evaluation.

When the effect of phallus length on the assignment recommendation was examined, it was found that in all three groups, phallus length was significantly higher in male assignments than in female assignments (Table 3).

According to the Prader classification with gender assignments recommendation, lower Prader stages (especially stage 1) were effective in making a female gender assignment in 46,XY DSD and sex chromosomal DSD cases. In addition, as the Prader stage increased, the decision-making ratio was gradually increased in favor of the male gender. However, the higher Prader stages were not associated with making a male gender assignment in 46,XX DSD cases. Moreover, the gender assignment of patients with Prader stage 1-4 was the female gender in a very large number of the 46,XX DSD cases. In general, it was found that a lower Prader stage was more effective in making a female gender assignment recommendation, than making a male gender assignment recommendation with a higher Prader stage (Table 4).

## Discussion

In this study, 20 years of experience in helping gender assignment, the causes and clinical characteristics of patients with DSD in a single referral clinic are presented. Gender assignment is always very difficult, complex and demands experience in the management of patients with DSD for both families and clinicians, particularly in cases where the gender appropriate for the clinical diagnosis is incompatible with the psychological gender of the patient. It should be recognized that every DSD is unique and has to be treated with individualized care. To our knowledge, this study has the longest timeframe, is the most comprehensive and has the largest number of cases in terms of gender assignment recommendation and assessing the factors affecting gender assignment from Turkey.

DSD are a heterogeneous group of conditions, which has an estimated incidence of 1:4500-5500 (10,11,12,33,34). In a recent study from Turkey by Aydin et al (35), it was found that the DSD newborn with ambiguous genitalia rate was 1.3/1000 newborns. However, this rate may be higher in our region where there is an increase in autosomal recessive forms of DSD due to higher rates of consanguinity, around 20% to 25% (35). This is in contrast to the consanguineous marriage rate reported by Aydin et al (35) (3 families of total 18 DSD patients). Nordenvall et al (36) remarked that the developmental anomalies of the external genitalia may be seen in 1:300 infants. However, not all of these conditions require gender assignment, including relatively common conditions such as isolated undescended testis and/or hypospadias.

Previous studies have reported a higher incidence of 46,XY DSD compared to 46,XX or sex chromosome DSD (35,37,38,39,40,41,42). In accordance with this the most common DSD group in our cohort was 46,XY DSD (50.9%). In a study with 117 patients from Thailand, it was reported that most of the cases were sex chromosome DSD (53%) (43). However, the majority of these patients were Turner syndrome. Girls with Turner syndrome were excluded from the present study because there is no necessity for gender assignment. Two Klinefelter syndrome patients were included because of ambiguous genitalia but other patients with Klinefelter syndrome without ambiguous genitalia, and thus without requirement for a gender assignment process were excluded.

Most patients with DSD are referred with ambiguous genitalia (35,37,38,39,43). In this study, ambiguous genitalia was the most common cause of admissions for all three DSD classifications (Table 1).

Despite the current advanced genetic analyses, a definitive genetic diagnosis can only be made in about 20% of cases of DSD (11,12,31,37). Compatible with this information, the rate of patients with undetermined causes of DSD was 18.6% (42/226) in our study. There were only two patients (2%) with undetermined causes in the 46,XX DSD group whereas this was 40/115 (34.7%) amongst the 46,XY DSD cases, thus constituting 40/42 (95%) of the patients without a definitive genetic diagnosis.

The etiologic cause of most of the patients with 46,XX DSD is CAH due to 21-OHD (37,38,39,44). In this study, CAH was the most common underlying etiological condition of 46,XX DSD (Table 1). CAH due to 21-OHD and 11-OHD accounted for 97.9% of 46,XX DSD in our series. Similarly, Ocal et al (39) found that 21-OHD and 11-OHD were the most frequent etiology (88.8%) of their 46,XX DSD group. De Paula et al (38) from Brazil with a 408 case series of genital ambiguity, Al-Mutair et al (45) from Saudi Arabia with a total of 120 DSD patients, and Al-Agha et al (46) from Australia report that the main etiology of 46,XX DSD was 21-OHD. However, Ganie et al (37) reported that the main referring cause of 46,XX DSD was ovotesticular in patients from sub-Saharan Africa.

It has been reported that only 50% of patients with 46,XY DSD can be given a definite diagnosis (44). In our study, the rate of 46,XY DSD patients with diagnosed causes was higher (n=75, 65.2%). The reason for this difference may be due to the further development of genetic understanding over the years. 5-alpha reductase deficiency was the most common etiology followed by CAIS and PAIS in 46,XY DSD (Table 1,2). The etiological distributions of both 46,XX DSD and 46,XY DSD patients were similar to previous studies (38,39,41,45, 46,47). Contrary to this, Ganie et al (37) report that the main etiological cause of 46,XY DSD was disorder of androgen synthesis or action.

Mixed gonadal dysgenesis was the most common etiology in the sex chromosome DSD group in our study (85.7%) which excluded Turner syndrome. Jaruratanasirikul and Engchaun (43) from Thailand reported that the most common sex chromosome DSD was Turner syndrome followed by Klinefelter syndrome and 45,X/46,XY DSD. Similar to this report, Ganie et al (37) from South Africa, with a total 346 cases diagnosed with DSD, noted that Turner syndrome constituted the largest proportion of the sex chromosome DSD group (61%), followed by mixed gonadal dysgenesis.

Gender identity is a characteristic which is influenced by various prenatal and postnatal variables. Psychosexual development plays an important role in the formation of sexual identity and is the main component of sexual identity, which is influenced by genetic status, pre/postnatal exposure to androgens, sociocultural factors, and family dynamics (6,39,48,49). Gender assignment is an important problem in DSD patients who have a virilized brain with undervirilized external genitalia (13,14,15,39).

Eleven of 97 46,XX patients (11.3%) had male gender orientation in the psychological evaluation, and were raised as the male gender by parents (nine were 21-OHD, one was 11-OHD, and one had Sertoli cell only syndrome; mean age of cases was 9.92±4.96 years). At the council meeting, six of these 11 cases were gender assignment recommendation male, two as female and three were not assigned and were recommended to be followed up.

Five of the patients who received a male assignment recommendation were 46,XX 21-OHD CAH and the other one was Sertoli cell only syndrome (Table 2). The mean age at presentation and at the time of the meeting of these five 21-OHD CAH patients was 7.56±5.26 years and 10.66±3.88 years, respectively. It was found that all of these patients were Prader stage 4-5, raised as male and their psychologic gender orientation was male, and all of the parents demanded a male gender assignment. The factors most strongly influencing recommended gender assignment in 46,XX cases included etiological diagnosis, age, psychologic gender and Prader staging (Table 2, 4).

Similar to our study, Khattab et al (13) report three 46,XX with 21-OHD CAH patients who were reared as male gender. In another study, of 50 DSD patients, 4/11 cases diagnosed with 46,XX DSD due to CAH had assumed a male social gender (15). This condition occurs due to prenatal and/or postnatal exposure to high levels of androgens that promote the masculinization of gender behavior (16,50). With the recent implementation of national neonatal CAH screening, it is hoped that late diagnosis of CAH, and therefore ambiguous genitalia, will be prevented.

For our council, the greatest difficulty in making gender assignment recommendations was in the 46,XY DSD group. The mean length of the phallus of patients who received a female assignment was 0.82±0.71 cm and 90% were Prader stage 1-2; etiological causes of these cases is shown in Table 2. Most of the 46,XY DSD patients who had no etiological diagnosis and had female gender assignment recommendations were Prader stage 1-2. Interestingly, psychological evaluation of these cases showed 8/9 had female gender and 1/9 had no gender orientation.

The majority, 93.7%, of the 46,XY cases with a male gender assignment recommendation and no etiological diagnosis were Prader stage 3-5. Moreover, 62.5% of these patients had no gender orientation yet. These findings suggest that, besides the etiologic diagnosis, the expectation of the family, phallus length and Prader stage were effective in the female assignment recommendations in 46,XY DSD cases. Furthermore, if there is no definite etiologic diagnosis, the most important factors in determining the gender assignment recommendation in 46,XY DSD patients were Prader stage and psychological gender orientation.

### Study Limitations

The major limitation of this study was the patients were only considered from presentation until the final decision for each individual by the gender assignment recommendation council. Due to ethical concerns, follow-up of patients after gender assignment recommendation was not included and thus there is no measure of agreement or discordance with the decision of the council reported.

## Conclusion

The most difficult aspect of managing a patient with DSD diagnosis who has ambiguous genitalia is the assignment of an appropriate gender. Specific diagnosis and psychological gender are more effective in gender assignment of DSD patients with an etiologic cause. Phallus length and Prader stage are important criteria in the gender assignment of patients with undiagnosed DSD. In this cohort none of the clinical, etiological or genetic features of the patients dominated the gender assignment decision. Gender assignment should be determined by evaluating the patient’s chromosome structure, specific diagnosis, fertility, Prader stage, phallus length, psychological orientation, family wish and the consensus opinion of experienced specialist physicians. Gender assignment becomes more difficult, especially if there is a mismatch of the gender the child is raised as, with the etiologic diagnosis. Gender assignment councils must have an experienced and multidisciplinary approach to the diagnosis, medical and/or surgical treatment, psychosocial support, and genetic counseling of patients with DSD. We hope that by publishing our extensive experience in this challenging clinical area we will help other clinicians and patients facing these difficult choices.

## Figures and Tables

**Table 1:**
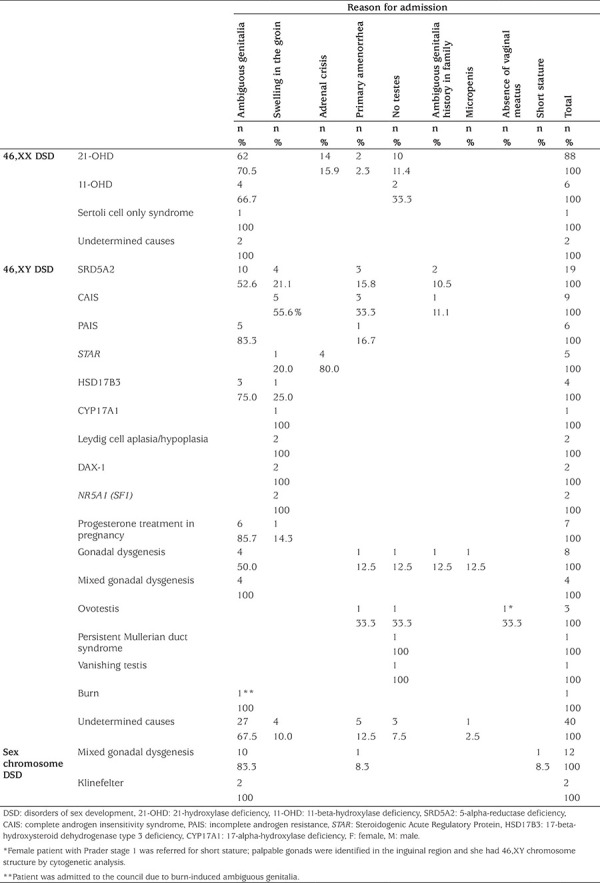
Distribution of admission reasons and etiological causes of patients

**Table 2:**
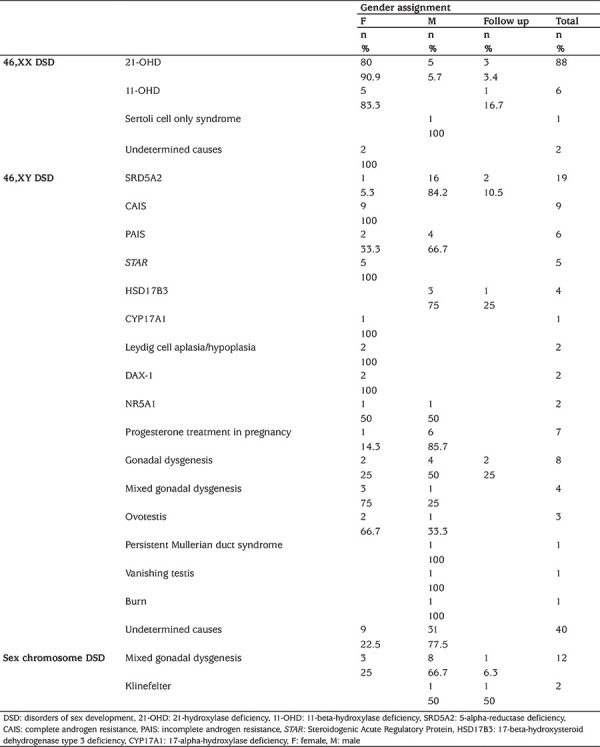
Etiological causes of disorders of sex development with gender assignment recommendations

**Table 3:**
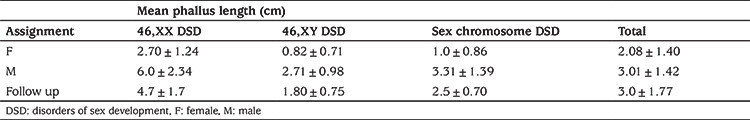
Evaluation of patients’ phallus length with gender assignment recommendations

**Table 4:**
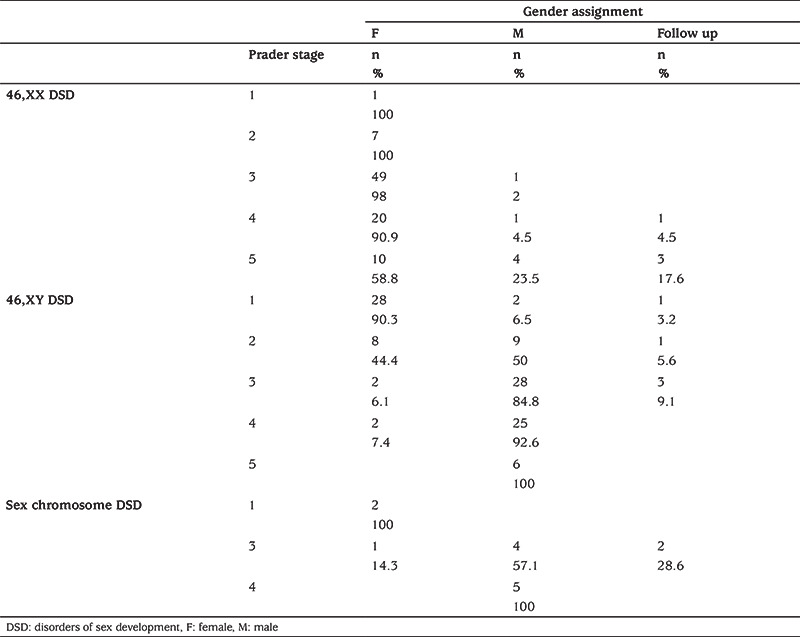
Prader classification with gender assignment recommendations
